# The Impact of Emotional Intelligence on Conditions of Trust among Leaders at the Kentucky Department for Public Health

**DOI:** 10.3389/fpubh.2015.00033

**Published:** 2015-03-13

**Authors:** Jennifer Redmond Knight, Heather M. Bush, William A. Mase, Martha Cornwell Riddell, Meng Liu, James W. Holsinger

**Affiliations:** ^1^Department of Health Management and Policy, College of Public Health, University of Kentucky, Lexington, KY, USA; ^2^Department of Biostatistics, College of Public Health, University of Kentucky, Lexington, KY, USA; ^3^Georgia Southern University, Statesboro, GA, USA; ^4^Department of Preventive Medicine, University of Kentucky, Lexington, KY, USA

**Keywords:** emotional intelligence, trust, leadership, public health workforce, stress management

## Abstract

There has been limited leadership research on emotional intelligence and trust in governmental public health settings. The purpose of this study was to identify and seek to understand the relationship between trust and elements of emotional intelligence, including stress management, at the Kentucky Department for Public Health (KDPH). The KDPH serves as Kentucky’s state governmental health department. KDPH is led by a Commissioner and composed of seven primary divisions and 25 branches within those divisions. The study was a non-randomized cross-sectional study utilizing electronic surveys that evaluated conditions of trust among staff members and emotional intelligence among supervisors. Pearson correlation coefficients and corresponding *p*-values are presented to provide the association between emotional intelligence scales and the conditions of trust. Significant positive correlations were observed between supervisors’ stress management and the staff members’ trust or perception of supervisors’ loyalty (*r* = 0.6, *p* = 0.01), integrity (*r* = 0.5, *p* = 0.03), receptivity (*r* = 0.6, *p* = 0.02), promise fulfillment (*r* = 0.6, *p* = 0.02), and availability (*r* = 0.5, *p* = 0.07). This research lays the foundation for emotional intelligence and trust research and leadership training in other governmental public health settings, such as local, other state, national, or international organizations. This original research provides metrics to assess the public health workforce with attention to organizational management and leadership constructs. The survey tools could be used in other governmental public health settings in order to develop tailored training opportunities related to emotional intelligence and trust organizations.

## Introduction

### Leadership and the public health workforce

The foundation of the public health infrastructure encompasses the information and knowledge systems, the public health workforce, and organizational capacity, which are required in order to accomplish core functions and essential public health services usually led by governmental public health organizations (1). The public health workforce has been described as the most important component of public health organizations and is the focus of 1 of the 10 essential public health services (2). Therefore, strengthening the public health workforce contributes to developing strong public health organizations and to improving the public health infrastructure (3). In order to determine how best to focus the limited resources available for improving the public health workforce, research that highlights and prioritizes the areas of greatest need within the workforce is important. According to the report, *The Future of the Public’s Health in the Twenty-First Century*, leadership training, support, and development should be prioritized in public health organizations (4).

In order to succeed in a complex public health environment, practitioners are required to have skills related to self-actualization, optimism, stress tolerance, happiness, and assertiveness, all of which have been shown to be associated with positive performance outcomes in the work place (5). Emotional intelligence and trust may be factors that predict organizational performance in public health settings as well as competencies that can be identified within organizations, promoted through training, and studied through organizational practice-based research (6). To date, the published literature on leadership, emotional intelligence, and trust has largely focused on the business and private sectors and not the public or governmental sectors.

### Emotional intelligence

Emotional intelligence is defined in several ways, and includes the ability to understand, perceive, and use emotions to enhance thought and relationships (7). The underlying causes of emotions such as context, challenges, communication, and community must be understood and considered in order for emotional intelligence competencies to be effectively exhibited (8). Emotional intelligence meets several standards of traditional intelligence: it can be seen as a specific mental ability that has “right” or “wrong” answers in its measure; it is correlated with other measures of intelligence, yet unique enough to be distinct; and it should develop with age (9).

Emotional intelligence is intuitively associated with leadership (10). One definition of effective leadership is, “the successful application of influence to the followers to achieve the leader’s and the group’s objectives” (11). Effective leadership is characterized, in part, by genuinely caring for people and enhancing positive feelings among followers (12, 13). Fambrough and Hart found that it is important that leaders consider the significance of emotions in their organizations (14). Leader-member relationships and emotional intelligence have also been studied. Emotional intelligence, authenticity, and relationship between the leader and the member may relate to leadership effectiveness (15).

Research conducted at the Center for Creative Leadership (CCL) evaluated leadership effectiveness utilizing “Benchmarks”^®^, a 360-degree leadership instrument that highlights skills related to leadership success. When leadership factors were compared with emotional intelligence factors among 236 leaders, 10 of the 16 leadership Benchmark factors were significantly related to the emotional intelligence factors (16). The more successful leaders had higher emotional intelligence subscale scores and of those subscales, 25% of the variance between successful and less-successful leaders could be attributed to: interpersonal relationships, stress tolerance, impulse control, and happiness (16). Unsuccessful leaders attributed their failure 11 percent of the time to technical incompetence and 23 percent of the time to lack of emotional intelligence (17). More specifically, low emotional intelligence is related to problems with interpersonal relationships, including difficulty changing or adapting that can negatively impact leaders, followers, peers, and others (18).

### Trust

Trust is a multi-faceted component, and the level of trust is related to the level of perceived risk. Greater trust is required when risk is higher. The characteristics of both the trustee and trustor are important for trust to exist (19). Leaders who act in a respectful and trusting manner, through honesty, fairness, and encouragement of teamwork, help to decrease stress and increase work performance (20). Trust has been shown to increase transparency, honesty, and openness related to information as well as admitting mistakes in order to create organizations that continue to remain viable (21).

Butler’s research focused on 11 conditions of trust that lead to trust in an individual based on the Leader-Member Exchange theory (LMX). The LMX focuses on the dyad relationship between the leader and the follower, making this a useful theory for studying the relationship between supervisors and the members of their staff (22). This research, as well as this review of other trust literature, demonstrated that: (1) trust is an important part of relationships; (2) trust is especially important for managers; (3) trust in a specific person and/or situation was more predictive than a general trust in others; and (4) in order to understand trust, there are conditions leading to trust that can be measured (22).

Other studies strengthen the concept that there are conditions that lead to trust. Several adjectives used to describe trust included: ability, benevolence, competence, consistent behavior, empowerment, encouragement, ethical practices, honesty, integrity, loyalty, openness, promise fulfillment, and respect, which also relate to Butler’s 11 conditions (19, 23–25).

### Emotional intelligence and trust

Emotional intelligence fosters trust which is increased by emotional intelligence (26). An outcome of good leadership (which requires emotional intelligence) is trust (25). Preliminary research indicates that emotional intelligence and trust are related to each other, which has been demonstrated in educational settings, in corporate and manufacturing settings, and in one local public health setting (25, 27–30). This current research assesses the concepts of emotional intelligence and trust based on a supervisor/staff member dyad in a state-level governmental public health setting.

### Study objective

The purpose of this study was to understand the relationship between aspects of emotional intelligence and conditions of trust between supervisors and the staff members who report to them in a public health setting.

## Materials and Methods

### Setting

The Kentucky Department for Public Health (KDPH) serves as the Commonwealth of Kentucky’s state health department. KDPH is led by a commissioner and is composed of seven primary divisions led by division directors reporting to the commissioner. Within the seven divisions, there are 25 branches led by branch managers who report directly to the division directors. Within each branch there are between 1 and 14 staff members who report directly to the branch managers. This structure provides leadership for the work of the state health department.

### Phases

This study consisted of two phases: Phase I was a feasibility study conducted with the commissioner and division directors to determine the best methods to be utilized in Phase II. Phase II was the full study conducted with branch managers and the staff members who reported to them. During Phase I, the participants completed the survey instruments proposed for Phase II and were asked for feedback on the process and instruments. Specifically, they were asked to comment on the process as well as provide recommendations or concerns related to the survey instruments. The primary lessons learned from Phase I included the following: (1) participants needed more than 2 weeks to participate; (2) participants should not be required to answer every question in the survey; and (3) participants should only be required to answer the question that is necessary to link the data from two surveys together.

### Participants

For Phase I, eight participants were eligible to participate; the seven division directors who reported to the commissioner and the commissioner. For Phase II, there were 24 active branch managers (one position was vacant) and there were a total of 149 staff members reporting to the branch managers (31). Thus, a total of 173 KDPH employees were eligible to participate in the Phase II (full) study.

### Instruments

The study utilized two survey instruments that were administered electronically: the first measured emotional intelligence and the second measured conditions of trust. The two survey tools selected for this study were the Emotional Quotient Inventory (EQ-i^®^; Reuven Bar-On) (32) for supervisors and the Conditions of Trust Inventory (CTI; John Butler) (22) for staff.

There are several measures of emotional intelligence found in the literature. The emotional intelligence models either focus on abilities, competencies, or a mix that focuses on personal factors (7, 33, 34). Researchers who endorse ability-based models believe that mixed-model approaches do not provide valid assessments because of the emphasis on self-reporting rather than ability testing through general and expert consensus (7). However, the self-reporting tests tend to have higher face and predictive validity than the ability-based models (35). The primary emotional intelligence tests included: Mayer-Salovey-Caruso Emotional Intelligence Test (MSCEIT; ability-based), Emotional Competence Inventory Version 2, (ECI-2; self-reporting), EQ-i^®^ (self-reporting), and the Emotional Intelligence Questionnaire (EIQ; self-reporting) (35).

The EQ-i^®^ was selected following a review of several critiques of emotional intelligence instruments as well as through personal communication with individuals who utilized such instruments at the University of Kentucky in leadership settings. The EQ-i^®^ focuses on a mix of personal factors and competencies. Permission was granted from Multi-Health Systems (MHS) to utilize the electronic version of the EQ-i^®^.

Trust relationships were evaluated in this study based on the LMX. The CTI was designed as a tool to increase understanding of the trust relationship between employees and managers. There are 11 supervisor behaviors considered in the CTI that facilitate trust including: (1) supervisor availability, (2) competence, (3) consistency, (4) discreetness, (5) fairness, (6) integrity, (7) loyalty, (8) openness, (9) promise fulfillment, (10) receptivity, and (11) overall trust (22). This survey instrument was selected to be administered to staff members since it provided a measure of trust and several components related to trust and because it had previously been utilized in research conducted within two local health departments; one in Northern Kentucky and the other in Cincinnati, OH, USA (36). The instrument developer provided a paper copy of the survey and written permission to use the survey in this research, including creating an electronic version of the survey.

### Scoring

The EQ-i^®^ was administered and scored through the MHS organizer web site, as a condition for utilizing the survey. The EQ-i^®^ scoring resulted in mean scores based on the self-reported answers to survey questions providing an overall emotional intelligence score, 5 scales and 15 subscales for each respondent. The five scales include: (1) intrapersonal, (2) interpersonal, (3) stress management, (4) adaptability, (5) general mood. Each of the 15 subscales fit within the 5 scales and include: (1) self-regard, (2) emotional self-awareness, (3) assertiveness, (4) independence, (5) self-actualization, (6) empathy, (7) social responsibility, (8) interpersonal relationships, (9) stress tolerance, (10) impulse control, (11) reality testing, (12) flexibility, (13) problem solving, (14) optimism, and (15) happiness (32).

The possible range of mean scores was <70–130+. Each participant received a mean score for overall emotional intelligence, each of the 5 scales and the 15 subscales. According to the technical manual, a score of <70 indicated markedly low emotional competencies and skills; 70–79 indicated very low emotional competencies and skills; 80–89 indicated low or underdeveloped emotional competencies and skills; 90–109 indicated average or adequate emotional competencies and skills; 110–119 indicated high or well-developed emotional competencies and skills; 120–129 indicated very high or extremely well-developed emotional competencies and skills; and 130+ indicated markedly high emotional competencies and skills (32).

The CTI was based on a Likert scale including 1 = strongly disagree, 2 = disagree, 3 = neutral, 4 = agree, and 5 = strongly agree (22). Each of the 11 conditions of trust subscales contained 1 negatively worded question to test for response pattern bias. These negatively stated inventory items were reverse coded during the data analysis stage. Reverse coding was performed based on previously conducted analysis by Mase (36).

### Validation

Both survey instruments have been previously validated. The EQ-i^®^ was the first scientifically validated test developed to measure emotional intelligence behavior (16). The test was first developed in order to explain which characteristics relate to positive psychological well-being and it has now developed into a tool that assesses the combination of emotional intelligence and psychological well-being (32). Test questions focus on the frequency and intensity with which an individual uses emotional and social skills (16), resulting in face and predictive ability with strong conceptual and theoretical underpinnings (16, 32, 35). The CTI has content, construct, convergent, and discriminant validity and is based on the LMX theory (22).

### Data collection

This research project was approved by the University of Kentucky Institutional Review Board under IRB Protocol No. 09-0764-X1B on October 14, 2009. This research was also approved by the Kentucky Cabinet for Health and Family Services Institutional Review Board Protocol #CHFS-IRB-DPH-FY10-45 on February 25, 2010.

The supervisors did not have access to their staff members’ trust scores, which protected the anonymity of staff members in order to encourage honesty in their assessment of their level of trust in their supervisor. This was especially important for some of the branches that only had two staff members, who could have been easily identified.

The study participants were recruited through face-to-face meetings where the research plan was presented and their participation requested. After these meetings, participants received electronic links to the surveys and follow-up reminders after 2 weeks to participate.

### Data management

The University of Kentucky Research and Data Management Center (UKRDMC) was responsible for housing, de-identifying, and linking the data. For Phase I, it received a copy of the commissioner’s name and division directors and provided unique identifiers for the division directors. For Phase II, it received a copy of the names of the branch managers, their branches, and the staff members who reported directly to them providing each participant with a unique identifier. The electronic version of the EQ-i^®^ required the participants to use their name in order to complete the survey.

An electronic version of the CTI was developed in cooperation with the UKRDMC. Study data were collected and managed using Research Electronic Data Capture (REDCap) tools hosted at the University of Kentucky (37). REDCap is a secure, web-based application designed to support data capture for research studies, providing: (1) an intuitive interface for validated data entry; (2) audit trails for tracking data manipulation and export procedures; (3) automated export procedures for seamless data downloads to common statistical packages; and (4) procedures for importing data from external sources. The CTI was administered to staff members directly reporting to the supervisors studied and required only the name of the branch in order for the survey to be completed. Each condition of trust score combined responses from four questions based on the analysis method of Mase (37). There were 10 staff members who did not have supervisors participate in the EQ-i^®^ and were excluded. Of those who were included, if any one of the questions was not answered, that respondent did not have a total condition of trust score for the variable being examined and was only excluded from the analysis of that variable. The following number of people were excluded from each of the conditions of trust measures: overall trust = 3; availability = 0; competency = 3; consistency = 2; discreetness = 5; fairness = 3; integrity = 2; loyalty = 1; openness = 2; promise fulfillment = 1; and receptivity = 0.

The UKRDMC received the results of the EQ-i^®^ as well as the CTI, linked the branch managers to the staff members who reported to them and de-identified the data. This data was then provided for analysis. Only the Phase II data was used in the analysis. Conditions of trust measures and staff characteristics were also provided in aggregate by supervisor. Conditions of trust measures and length of service aggregate measures were calculated using means; percentages were used for gender; and counts were used for total number of employees supervised.

### Statistical analysis

The primary measures of interest were 5 emotional intelligence scales for supervisors and their relationship with 11 conditions of trust aggregate scores. The five emotional intelligence scales were intrapersonal, interpersonal, adaptability, stress management, and general mood. The conditions of trust measures collected from staff included scores on overall trust, availability, competency, consistency, discreetness, fairness, integrity, loyalty, openness, promise fulfillment, and receptivity and each variable was averaged for each supervisor. These were summarized with descriptive statistics (*n*, median, first and third quartiles, and interquartile ranges), overall, and by supervisor gender. Other variables of interest included the gender of the supervisor, the number of staff per supervisor, female staff percentage as well as average staff service years in KDPH, current branch, and the field of public health. Continuous variables were summarized with descriptive statistics and categorical variables with counts and percentages. Pearson correlation coefficients and corresponding *p*-values are presented with scatterplots to provide the association between emotional intelligence scales and the conditions of trust.

## Results

The response rate for the full study was 79% (19/24) for supervisors (branch managers) and was 65% for (98/149) staff members who reported directly to the supervisors. In total, there were 16 supervisors who completed the EQ-i^®^ and had staff members participate in the CTI. Eighty-eight staff members completed CTI who had supervisors complete the EQ-i^®^. Female supervisors accounted for 56.25% of participants (Table 1). Supervisors varied in the number of staff reporting to them (2–11) with a median of 6.2 average service years for staff in KDPH, 3.9 average years in current branches, and 9.7 average years in the field of public health. Minimal differences were observed when service years were stratified by supervisors’ gender.

**Table 1:** **Demographics by supervisor**.

	All	Female	Male
Supervisor (%)	16	9 (56.25%)	7 (43.75%)
Number of staff by supervisor	5 (2,11)	5 (2,11)	5 (2,10)
Female staff average proportion	0.9 (0.4,1.0)	0.9 (0.4,1.0)	0.9 (0.6,1.0)
**Staff service average years**
KDPH^a^	6.2 (2.8,9.6)	6.1 (2.8,9.6)	6.2 (3.4,9.6)
Branch^b^	3.9 (1.7,9.2)	4.2 (1.2,7.6)	3.4 (2.2,9.2)
Public health^c^	9.7 (1.2,16.8)	10.0 (1.2,15.0)	9.4 (5.8,16.8)

*Summaries are presented as n (%) or median (min, max)*.

The average number of years of service for staff was calculated by supervisors and summarized for

*^a^KDPH*,

^b^Branch, and

*^c^Public health*.

The median EQ scores were similar for male and female supervisors and remained in the average/adequate range for each major group (Table 2). However, male supervisors tended to have more variability in responses than female supervisors. When compared within each subgroup, the highest median score for male supervisors was stress tolerance and the lowest were self-actualization and empathy. The highest observed median score for female supervisors was empathy and the lowest was self-regard. Male supervisors had noticeably higher median scores than female supervisors in terms of self-regard, independence, and happiness, while female supervisors’ median scores were observed to be higher with respect to empathy, self-actualization, and impulse control. The lowest observed median score for males was the highest median score for females (empathy).

**Table 2:** **Emotional intelligence scores for supervisors**.

EQ scores (*n*)	All (16)	Male [7 (43.75%)]	Female [9 (56.25%)]
Total EQ	111 (104,117)	111 (100,118)	108 (107,116)
Intrapersonal	109 (104,117)	109 (95,126)	108 (107,116)
Self-regard	107 (97,113)	110 (95,113)	100 (99,112)
Emotional self-awareness	111 (105,118)	108 (92,116)	114 (106,118)
Assertiveness	113 (108,118)	116 (108,127)	111 (108,117)
Independence	107 (100,115)	114 (95,123)	106 (104,113)
Self-actualization	106 (100,110)	101 (95,118)	107 (105,110)
Interpersonal	107 (98,113)	108 (94,113)	106 (102,112)
Empathy	103 (92,117)	101 (85,105)	116 (100,118)
Social responsibility	108 (103,112)	108 (100,110)	109 (104,112)
Interpersonal relationship	103 (99,110)	103 (96,117)	103 (99,110)
Adaptability	110 (103,114)	108 (101,113)	111 (105,118)
Reality testing	112 (106,116)	107 (104,112)	113 (112,118)
Flexibility	109 (100,116)	106 (94,115)	110 (104,116)
Problem solving	105 (99,109)	105 (105,114)	105 (95,108)
Stress management	113 (108,116)	112 (104,116)	114 (108,116)
Stress tolerance	115 (108,118)	118 (108,118)	114 (108,116)
Impulse control	109 (101,115)	103 (100,117)	110 (106,113)
General mood	106 (101,112)	105 (99,114)	106 (102,110)
Happiness	106 (101,113)	112 (97,114)	104 (102,109)
Optimism	107 (99,111)	104 (96,110)	107 (104,111)

Overall, the median aggregated condition of trust scores ranged from 3.8 (openness) to 4.5 (availability and competency; Table 3). For male supervisors, the highest observed median condition of trust score (*M* = 4.5) was receptivity and the lowest (*M* = 3.8) were discreetness, fairness, loyalty, and openness. Among female supervisors, the highest median condition of trust score (*M* = 4.8) was fairness, which was one of the lowest observed scores for male supervisors. In general, female supervisors tended to have higher observed median aggregate condition of trust scores than male supervisors in all domains except receptivity.

**Table 3:** **Aggregated staff conditions of trust scores for supervisors by gender**.

Condition	All	Male	Female
Overall trust	4.0 (3.3,5.0)	4.0 (3.4,5.0)	4.0 (2.8,5.0)
Availability	4.5 (3.8,5.0)	4.3 (4.0,5.0)	4.5 (3.8,5.0)
Competency	4.5 (3.5,5.0)	4.3 (3.8,5.0)	4.7 (3.3,5.0)
Consistency	4.0 (3.0,4.5)	4.0 (3.3,4.5)	4.0 (3.0,4.5)
Discreetness	4.0 (3.0,5.0)	3.8 (3.0,4.8)	4.4 (3.0,5.0)
Fairness	4.0 (3.5,5.0)	3.8 (3.5,5.0)	4.8 (3.3,5.0)
Integrity	4.3 (3.3,5.0)	4.0 (3.3,5.0)	4.5 (2.8,5.0)
Loyalty	4.0 (3.0,5.0)	3.8 (3.0,4.8)	4.3 (2.5,5.0)
Openness	3.8 (3.0,4.3)	3.8 (3.1,4.0)	4.0 (3.0,4.5)
Promise fulfillment	4.0 (3.0,5.0)	4.0 (3.0,4.8)	4.0 (3.0,5.0)
Receptivity	4.1 (3.5,5.0)	4.5 (3.5,5.0)	4.0 (3.3,5.0)

*Staff scores were averaged for a supervisor and these averaged values are summarized here for supervisors (n = 16) as median (Q1, Q3)*.

**Figure 1:**
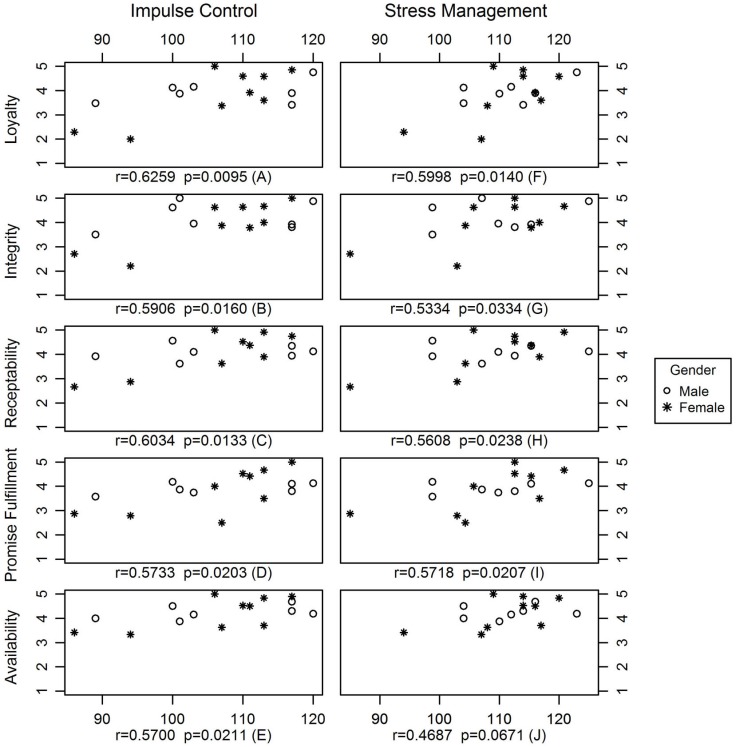
**Correlation between stress management and conditions of trust and between impulse control and conditions of trust**.

Significant positive correlations were observed between supervisors’ stress management (EQ-i) and the aggregate measures of trust, loyalty (*r* = 0.6, *p* = 0.01), integrity (*r* = 0.5, *p* = 0.03), receptivity (*r* = 0.6, *p* = 0.02), promise fulfillment (*r* = 0.6, *p* = 0.02), and availability (*r* = 0.5, *p* = 0.07). This analysis was further performed using the specific EQ-i stress subscales: impulse control and stress tolerance (Figure 1). There was no significant correlation between stress tolerance and any of the aggregated staff conditions of trust. However, significant positive correlation was found between impulse control and loyalty (*r* = 0.6, *p* = 0.01), integrity (*r* = 0.6, *p* = 0.02), receptivity (*r* = 0.6, *p* = 0.01), promise fulfillment (*r* = 0.6, *p* = 0.02), and availability (*r* = 0.6, *p* = 0.02).

## Discussion

In order to assure a competent public health workforce, continuing to identify and prioritize areas related to public health leadership and training is crucial. The Public Health Workforce Research Agenda includes two areas that are particularly applicable to this research: (1) how is workforce competency measured at an individual level or for a specific role such as leadership? and (2) to achieve a significant impact, are certain individual competencies of greater importance than others? (38).

The finding that supervisors have average or high levels of emotional intelligence is important data for the KDPH. Since the supervisors have a higher than average emotional capacity to deal with stress and since stress management has an impact on conditions of trust, there are implications for recruitment and retention in leadership positions for public health.

Recruitment and retention are priorities identified by the Centers for Disease Control and Prevention Office of Workforce and Career Development (39). Perhaps the participating supervisors developed the ability to effectively manage stress prior to assuming a leadership position or they may have developed this ability while working within a stressful environment. Turnover rates may be higher among public health managers who do not have a high capacity to manage stress which may have an impact on the staff member’s ability to trust the supervisor, potentially leading to staff member turnover. In this study, information was not collected from the supervisors related to their length of service in public health, in their current branch, or in KDPH. Future workforce-related research should investigate the relationship between stress management, length of service, and propensity toward retention or turnover. In addition, research could focus on the public health organization as the unit of analysis, providing important environment and contextual information.

Other settings tend to have emotional intelligence rates that are similar to the ones found at KDPH. A group known as the Young President’s organization, which includes individuals who have become top leaders and earned a minimum of $5 million dollars by the age of 39, had stress tolerance, with a mean of 109, among their highest scores (32). An assessment within the financial services industry found above average emotional intelligence scores, with a stress tolerance score of 105 (32). The mean scores of stress tolerance at KDPH were even higher at a mean of 111, demonstrating capacity for strong leadership and stress tolerance even beyond these private industries.

There are implications for training and continuing education related to leadership, emotional intelligence, and trust in KDPH. Even though stress management ranks consistently high among supervisors, since there is a strong relationship between stress management and several conditions of trust, ongoing training and professional development related to stress management should be conducted to enhance an environment of trust and enhance recruitment and retention within public health settings.

Public health leaders should also consider targeted training approaches to continuing education opportunities. As a benefit for supervisors to participate in this study, the principal investigator partnered with a certified EQ-i^®^ administrator from the Kentucky Public Health Leadership Institute who downloaded individual resource reports and provided personal results feedback to interested participants as an opportunity for professional development and coaching. The study findings indicate the need to consider tailoring training opportunities to areas of greatest improvement for certain demographic groups in the public health workforce, which could involve focusing on employees by gender, race, or seniority. Training opportunities could be piloted in certain branches or divisions rather than Department-wide.

Certain limitations should be considered related to this study. The research design was a non-randomized cross-sectional study; therefore results may not be generalizable outside the study population. However, since all the state health department branch managers and the staff members who reported directly to them were invited to participate, the results may be generalized to other state governmental public health settings with similar structures. There was a small sample size (16 supervisors) and there were average and high emotional intelligence scores. The only correlation between emotional intelligence and any conditions of trust was in stress management as well as one of the subscales of stress management, impulse control. The study was based on self-reported data using electronic survey tools, which could introduce recall and perception bias. Another limiting factor relates to participation. There may be differences between those who participated and those who did not participate. Also, the study does not include the organizational context or past examples of relationships and triggers that may have an impact on the results (40).

This research lays the foundation for emotional intelligence and trust to be assessed further in public health settings. The principal investigator shared the results of the study with the KDPH through small group meetings with the commissioner and with supervisors and staff members. The KDPH employees demonstrated an interest in further studies examining these relationships in other leaders. For instance, this study measured the relationship between branch managers and the staff members who report to them, but it did not look at the relationship between the branch manager and the division director. Several staff asked if it would be possible to examine these other relationships in future research. Utilizing the KDPH leadership staff and implementing two validated surveys provides a methodology that can be replicated within the KDPH as well as with other public health settings.

## Conflict of Interest Statement

The authors declare that the research was conducted in the absence of any commercial or financial relationships that could be construed as a potential conflict of interest.
